# Population pharmacokinetic model selection assisted by machine learning

**DOI:** 10.1007/s10928-021-09793-6

**Published:** 2021-10-27

**Authors:** Emeric Sibieude, Akash Khandelwal, Pascal Girard, Jan S. Hesthaven, Nadia Terranova

**Affiliations:** 1 School of Basic Sciences, EPFL, Lausanne, Switzerland; 2 Merck Institute for Pharmacometrics (an affiliate of Merck KGaA, Darmstadt, Germany), Lausanne, Switzerland; 3 Merck KGaA, Darmstadt, Germany; 4 Chair of Computational Mathematics and Simulation Science (MCSS), Ecole Polytechnique Fédérale de Lausanne (EPFL), Lausanne, Switzerland

**Keywords:** Deep learning, Genetic algorithm, Model-informed drug discovery and development, Neural network, Pharmacometrics, Population PK/PD

## Abstract

A fit-for-purpose structural and statistical model is the first major requirement in population pharmacometric model development. In this manuscript we discuss how this complex and computationally intensive task could benefit from supervised machine learning algorithms. We compared the classical pharmacometric approach with two machine learning methods, genetic algorithm and neural networks, in different scenarios based on simulated pharmacokinetic data. Genetic algorithm performance was assessed using a fitness function based on log-likelihood, whilst neural networks were trained using mean square error or binary cross-entropy loss. Machine learning provided a selection based only on statistical rules and achieved accurate selection. The minimization process of genetic algorithm was successful at allowing the algorithm to select plausible models. Neural network classification tasks achieved the most accurate results. Neural network regression tasks were less precise than neural network classification and genetic algorithm methods. The computational gain obtained by using machine learning was substantial, especially in the case of neural networks. We demonstrated that machine learning methods can greatly increase the efficiency of pharmacokinetic population model selection in case of large datasets or complex models requiring long run-times. Our results suggest that machine learning approaches can achieve a first fast selection of models which can be followed by more conventional pharmacometric approaches.

**Supplementary Information:**

The online version contains supplementary material available at 10.1007/s10928-021-09793-6.

## Introduction

Model-informed drug discovery and development (MID3) is a process which applies quantitative modeling to pre-clinical and clinical data to accelerate and optimize drug development [1]. MID3 plays a key role at each stage of drug development by quantifying the risk–benefit ratio of the treatment in the general population and in sub-populations, therefore increasing confidence in decision-making and reducing development costs [2].

MID3 has a large range of applications, including characterizing the drug concentration-pharmacodynamic (PD) response relationships [3], explaining drug variability by identifying clinically relevant factors which impact on desired outcomes [4], and predicting the consequences of formulation changes on drug performance [5].

Among the techniques available in MID3, population modeling is a tool which describes the relationships between patients´ physiological characteristics and model parameters governing drug concentrations, or drug response and their distribution across a population [6]. Population pharmacokinetic (PK) and PD models are used to describe relationships between a dependent variable (e.g., concentration or response) and an independent variable (e.g., time). These models are also used to investigate sources of variability [7]. Population models favor statistical (nonlinear) mixed effect modeling techniques. This methodology allows the development of models containing both fixed and random effects.

Nonlinear mixed effects modeling (NONMEM) was historically developed to build population PK models around a first order approximation of the random effect. NONMEM allows computing a model’s likelihood for a given dataset, the main difficulty of this model type. Given the estimation bias produced by this method, this approach was further enriched with a variety of other algorithms borrowed from the statistical literature, including first order conditional estimate, Gaussian Quadrature and Laplacian, Gibbs sampling and Metropolis Hasting for full Bayesian modeling, stochastic approximation of expectation–maximization algorithm (SAEM [8]), and others [9]. Another powerful program for developing population models is Monolix, which was initially developed around the SAEM algorithm [10].

At present, model selection is achieved using specific metrics and expert decisions based on clinical and biological relevance. In the age of digital medicine, where large amounts of data are available, models have become increasingly complex, and model selection can be further optimized using recent state-of-the-art developments in artificial intelligence algorithms.

In addition to existing approaches to model selection, machine learning (ML) and deep learning (DL) offer numerous algorithms which can be applied to medicine [11–13] and drug development [14–20] when built on statistical rules. ML and DL allow researchers to make accurate predictions and can handle large datasets, suggesting a potential for them to be used in population modeling. Indeed, their successful use in population modeling has been recently demonstrated, with fast and efficient screening of covariates performed in large datasets and complex models [21].

One existing ML approach for model selection is genetic algorithm (GA), an optimization process that tries to mimic Darwinian natural selection [22]. GA has been applied to solve many discrete optimization problems where performing exhaustive research is not possible or when little prior knowledge is available [23, 24].

Neural networks (NNs) are another existing ML approach for model selection belonging to a group of supervised learning algorithms. These learn by processing training examples and adjusting their associations according to a learning rule, then minimizing the selected cost function until the produced output is increasingly similar to the target output [25].

In this study we investigated the accuracy and computational costs for ML approaches (GA and NNs) and classical pharmacometric (PMX) approaches in the context of population PK model selection.

## Methods

### Library of models

To simulate then subsequently estimate a model with Monolix, the structural model was first implemented into MlxTran. Starting from the existing Monolix model library composed of 36 models, additional models were created which allowed alternative features for administration route (e.g., intravenous, oral, or subcutaneous), absorption (e.g., first [1] and zero [0] order, with and without lag time [T_lag_] or transitory compartments), elimination (e.g., linear or Michaelis–Menten), and numbers of compartments (up to three). These derived models were further combined with four different residual error models, defined as follows:

1. Constant: $${C}_{obs}= {C}_{pred}+a\bullet \epsilon$$
2. Proportional: $${C}_{obs}= {C}_{pred}+{C}_{pred}\bullet b\bullet \epsilon$$
3. Combined 1: $${C}_{obs}= {C}_{pred}+({C}_{pred}\bullet b+a)\bullet \epsilon$$
4. Combined 2: $${C}_{obs}= {C}_{pred}+\sqrt{({C}_{pred}\bullet b+a)}\bullet \epsilon$$

where *C*_*obs*_ was the measured concentration, *C*_*pred*_ was the predicted concentration, *a* and *b* were constants to be optimized, and *ε* was a random variable normal.

Overall, our library comprised of 504 (126 structural × 4 error models) different structural + error models (Table 1). On top of these, assessments of potential covariance matrices of random effects describing between-patient variability and investigations of their distributions (among normal, lognormal, and probit) were performed. Thus, by adding combinations of these to each of the 504 models, the total number of tested models was much larger and included investigations of statistical models. Explorations of levels of random effects above between-patient was not in scope of this work.

**Table 1:** Main features (structural + residual error) of models in the considered library approaches

Input function	# compartments	Enterohepatic circulation	Output function	Residual error
Bolus 0 order 0 order + T_lag_ 0 order 1 order + T_lag_ 1 order + transitory compartment 0 order + 1 order	1 2 3	No Yes	Linear Michaelis–Menten Linear + Michaelis–Menten	Additive Proportional Combined 1 Combined 2

*T*_*lag*_ lag time

### Method performance

The software Simulx and the MLXTRAN library of models were used to simulate PK profiles which were then fitted to a panel of models in Monolix (MonolixSuite2018R2; Lixoft, Antony, France). The performance of different methods was assessed by their ability to recover true structural (Table 2) and statistical models selected to have a variety of models, which differed in terms of input model, number of compartments, and output model. Statistical assumptions and relationships (e.g., parameter distributions, covariance matrix structure for random effects, and residual error model) also differed across models.

**Table 2:** Summary of simulated datasets investigated using PMX and GA approaches

Dataset	Input model	# compartments	Output model	Error model
Dataset 1	Transit compartment + 1 order	1	Linear	Proportional
Dataset 2	First order + 0 order	1	Linear	Combined 1
Dataset 3	Bolus	2	Michaelis–Menten + linear	Combined 1
Dataset 4	T_lag_ + 1st order	2	Linear	Additive
Dataset 5	Bolus	3	Linear	Combined 1

*GA* genetic algorithm, *PMX* pharmacometric, *T*_*lag*_ lag time

To characterize the analysis workflow for this study, two paths were selected: one to reflect the automatic execution of PMX and GA approaches, and one to reflect NN tasks. The NN task was conducted using Python 3.7 along with Pytorch (1.3.1) installed on Anaconda 1.9.12.

**Fig. 1:**
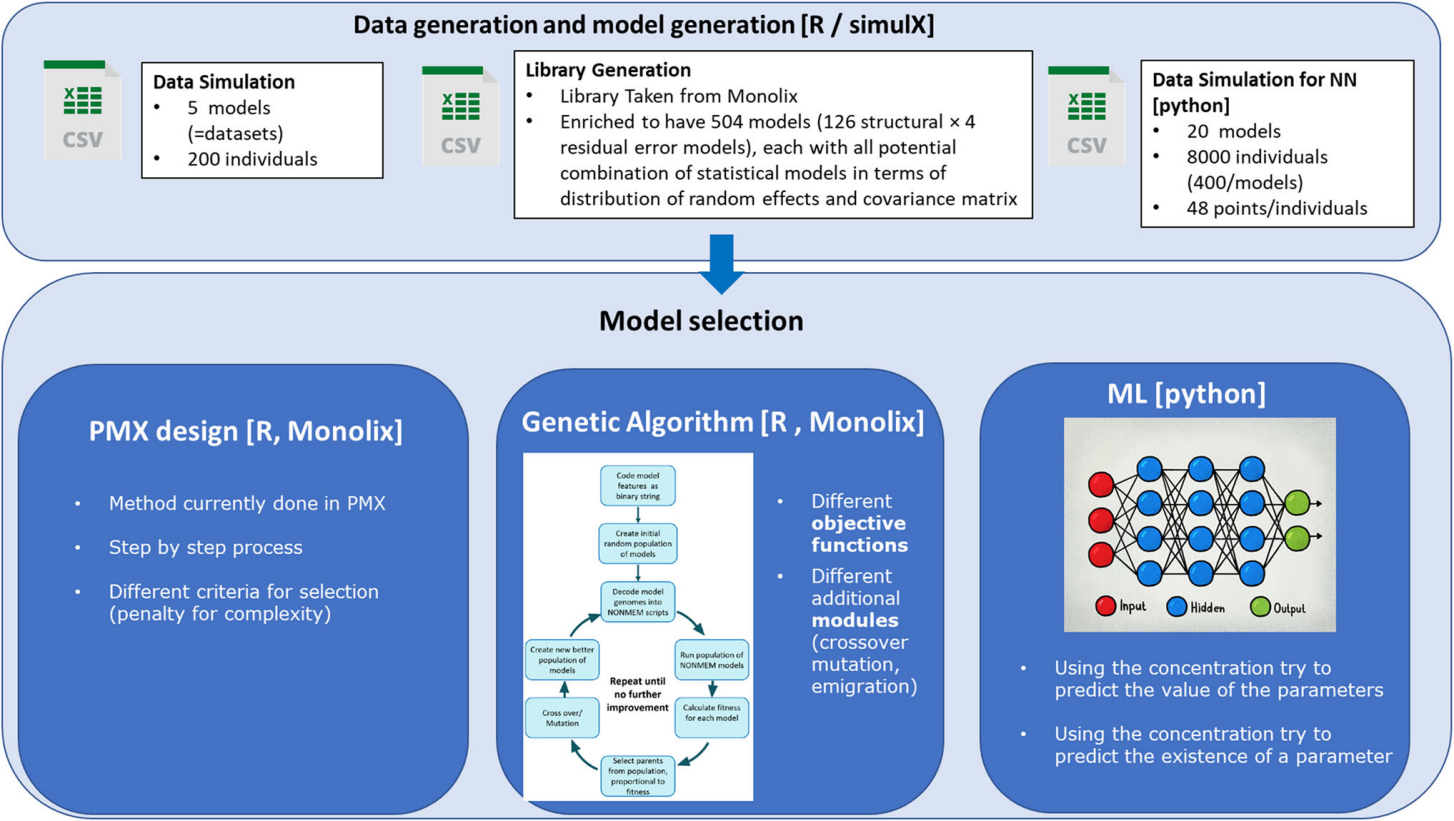
General workflow—the first step was the data and model library generation, followed by the investigation of the three approaches selected (PMX, GA, and NN). *GA* genetic algorithm, *NN* neural network, *PMX* pharmacometric

As shown in Fig. 1, the first two approaches were automatically executed by:

1. Simulating a dataset based on a model from the library
2. Running the model in Monolix
3. Evaluating the results according to PMX criteria or GA fitness in R version 3.5.1 [26].

These three steps were repeated until the best model was found.

PMX and GA were assessed on five generated datasets (Online Resource 1). For NN tasks, a learning phase was required and implemented by training the NN on a large training set having simulated concentration profiles as input and model parameters, included as values for regression and binary labels for classification, as output. Then, the NN model was fit and its performance assessed on independent test sets (Online Resource 1).

### PMX model building

PMX model building is a step-by-step process towards a “fit-for-purpose” model [6, 7]. For this work, the process was implemented in R version 3.5.1 by automatically executing different structural and statistical models through Monolix, then comparing results to commonly pre-defined model selection criteria also considered in the GA fitness function. After selecting the structural model, the covariance matrix of random effects was built starting from a diagonal matrix and progressively assessing the significance of correlation terms. Finally, an automatic exhaustive search on the commonly used error models was performed to characterize the residual unexplained variability [27].

### Genetic algorithm for model selection

**Fig. 2:**
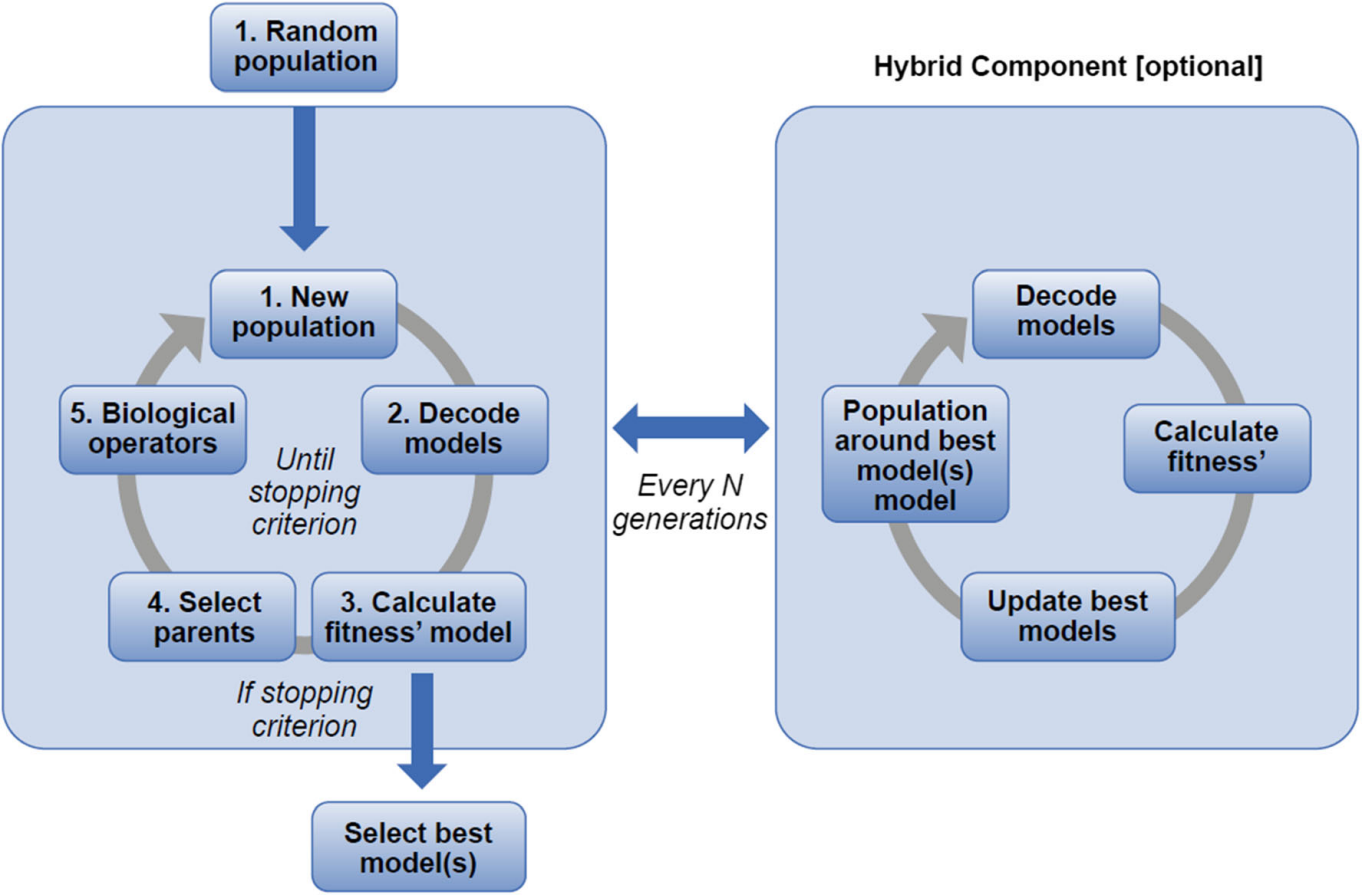
Hybrid GA—the hybrid component makes the GA convergence faster by performing an exhaustive search around the best models. *GA* genetic algorithm. *N* is a parameter (integer) set by the user for the GA

GA is a search heuristic inspired by Charles Darwin’s theory of natural selection. As shown in Fig. 2, by starting from a random population of models, the GA repeatedly modifies a population with a “natural selection” occurring at each generation. Over successive generations, the population “evolves” towards an optimal solution. The addition of a hybrid component supports a local search procedure for a faster convergence to the best model. The application of GA to PK model selection problems follows the same rationale [23], with populations made of PK models selected in subsequent generations according to a fitness function, based on pre-defined PMX criteria. In our study, models were estimated in Monolix using a SAEM algorithm.

#### Model representation

To increase parallels with natural selection, each PK model was represented as chromosomes with eight genes as binary representations of the different model components. The structural and error models were first represented by five genes: absorption (seven levels), circulation (two levels), compartments (three levels), elimination (three levels), and error (four levels). Components with more than two levels were encoded into multi-level binary representations. The remaining three genes encoded the covariance matrix. This gene-based model representation ensured that any structural model could be uniquely encrypted into a sequence of bits. The initial population of PK models was obtained by first randomly generating a population of structural and error models, then assigning a statistical model for the between-patient variability to each of them. An example for a population of four chromosomes is shown in Online Resource 2, and an example of a model population of size 3 is shown in Table 3.

**Table 3:** Example of generated population of size 3 in GA for A structural and residual error genes, and B statistical models for random effects

Model	Absorption	Circulation	Compartments	Elimination	Error
A					
1	001 (0 order)	0 (no)	10 (3 comp)	00 (linear)	01 (proportional)
2	011 (1 order)	0 (no)	01 (2 comp)	10 (mixed)	00 (constant)
3	000 (Bolus)	1 (yes)	00 (1 comp)	01 (Michaelis–Menten)	11 (combined 1)

Model	Variability	Correlation	Distribution
B			
1	TK0, V, Q2, V3, CL	CL, V, V3	CL, V, V3, V2: lognormal, TK0: normal
2	Ka, V, Km and Vm	Ka, V	Ka, Km: lognormal, V, Vm: normal
3	Not present	NA	NA

*CL* clearance, *GA* genetic algorithm, *Ka* 1 order absorption, *Km and Vm* Michaelis–Menten elimination, *PK* pharmacokinetic *Q2* inter-compartmental clearance, *TK0* 0 order absorption, *T*_*lag*_ lag time, *V* volume for central compartment, *V2* volume for second compartment, *V3* volume for third compartment. Parameters depend on the generated structural model

For parameters with assigned between-patient variability, the distribution of random effects was randomly chosen among normal, lognormal, and probit. Similarly, correlations between parameters with variability were randomly set in the covariance matrix.

#### Model selection

The selection of models (called parents) that contributed to the population at the next generation was performed according to tournament selection [28]. This method consists of running several “tournaments” among a few models, assessing each of them against a random opponent and selecting the winner (the one with the best fitness) for crossover in the next generation.

The first advantage of tournament selection is robustness. Moreover, it also ensures stability in the population size, as there is one game for each model and the size of the population does not change from one generation to another. The second advantage is that the best model is always selected to be one parent of the next generation. Online Resource 3 is an example of a population of four models, where the goodness of a model was proportional to its fitness value. In this example, a random opponent was randomly assigned to each of the four models, and the winners selected to be parents in the next generation.

#### Population evolution

Following the selection of parents, random couples are created to generate children through crossover and mutations, as in biological reproduction [29]. With the crossover, two children are created by combining different parts of the parents. In our application, combined parts involved the structural and statistical model (i.e., the covariance matrix of random effects), which was strictly dependent on the presence or absence of certain parameters in the structural model. For this reason, random effects and correlation between random effects were inherited from the respective parameters’ parent, with deletion of terms associated with parameters no longer present in the model. In other words, in case of conflicts (i.e., when a parameter was present in the two-parent models with two different distributions), child 1 received the distribution from parent 1 and child 2 from parent 2. Online Resource 4 presents an example of such crossover.

Random changes in genes could occur independently with the same defined probability. A third function called immigration could also be applied before the crossover by deleting the worst parents from the population and replacing them with randomly generated models. This option is useful to avoid being stuck in a local minimum and to increase the speed of convergence since these parents may be non-optimal.

The GA process continued over successive generations until a stopping criterion, here based on a pre-defined number of generations, was reached and the best model (or the *k* best models for some problems) of the last generation was given as output.

#### Fitness function

A key element of population evolution towards a satisfactory model is the choice of the fitness function. In this study, the fitness function was close to the one presented by Bies et al. [23]. It was based on the objective function value given by Monolix and a penalty term added for (i) the number of parameters, (ii) parameter correlation values > 0*.*95, (iii) failed convergence, and (iv) missing covariance step. The following two fitness functions were tested, with the second including a penalty for shrinkage values on random effects > 0.7:

1. *F*1 =  − 2 · *Log-likelihood* + 10.83· #*parameters* + 400 · 1*nonconvergence* + 100 · 1*correlation* > 0*.*95 + 100 · 1_CovarianceStep_
2. *F*2 =  − 2 · *Log-likelihood* + 10*.*83 · #*parameters* + 400 · 1*nonconvergence* + 100 · 1*correlation* > 0*.*95 + 100 · 1_CovarianceStep_ + 100 · 1_Shrinkage>0.7_

The penalty value of 10*.*83 for a new parameter was equivalent to performing a likelihood ratio test and accepting the new parameter if the p-value was < 0*.*001.

#### Hybrid component

The GA is known to quickly converge in an optimal area, but it can take time to make minor changes in the model to obtain the best result [30]. A hybrid GA may be used to tackle this drawback and increase the speed of convergence (see Fig. 2) [31]. The hybrid component performed an exhaustive local search around the best models every *N* generation (a parameter [integer] set by the user for the GA). This was achieved by generating a new population of all the possible models with a change of one bit from the best model. These models were then estimated and if some changes led to a better model, the best model was updated accordingly (i.e., the corresponding gene in the chromosome was updated). In practice, more than one model could be selected, and an exhaustive search was done around a pre-defined number (n = 2 in this study) of models. Algorithms using this component are known as hybrid GA.

### Neural network for model prediction and selection

**Fig. 3:**
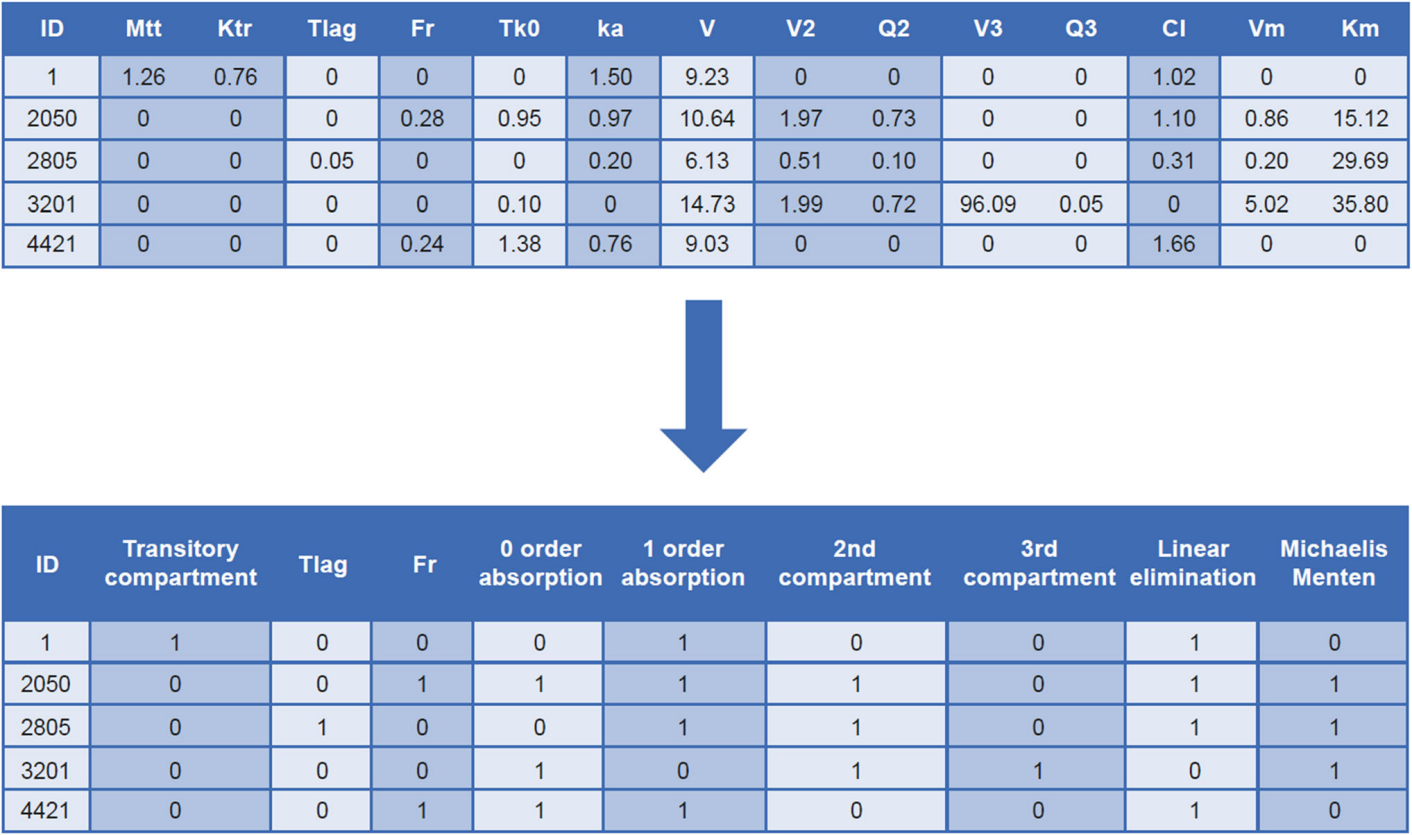
Example of training output parameters for the two NN tasks. *CL* clearance, *Fr* bioavailability, *Ka* 1 order absorption, *Km and Vm* Michaelis–Menten elimination, *Ktr* transitory compartment, *Mtt* transitory compartment, *NN* neural network, *Q2 and Q3* inter-compartmental clearance, *TK0* 0 order absorption, *T*_*lag*_ lag time, *V* volume for central compartment, *V2* volume for second compartment, *V3* volume for third compartment. *Note* For the regression task, individual pharmacokinetic parameters constitute the output to be predicted (top table). Data for the classification task can be derived from this by combining parameters into model components binarily labeled according to their presence or absence (bottom table)

In our application, NN training sets were constructed and continuously enriched with simulated data using models and PK properties commonly observed across developed and approved drugs. An example of training parameters coding for the two NN tasks, regression and classification, is presented in Fig. 3. The full dataset in this study comprised of concentration profiles from 8000 virtual individuals generated according to 20 different structural models (Online Resource 1). From this, data from 1600 randomly selected individuals were removed from the learning phase and used for the test set. Two different approaches were investigated for model selection: regression and classification.

For the regression task, individual PK parameters were recorded for the output. The value of 14 parameters (transitory compartment [Mtt, Ktr], T_lag_, bioavailability [Fr], 0 order absorption [Tk0], 1 order absorption [ka], volume for central, second and third compartment [V, V2, and V3], clearance and inter-compartmental clearance [CL, Q2, and Q3], and Michaelis–Menten elimination [Vm and Km] were predicted. For predicted values close to 0, parameters were removed thus informing the model selection. The mean square error (MSE) was used as the cost function with the ADAM optimizer and ELU activation function. L2 regularization with different penalty was also used. A grid search was performed as summarized in Online Resource 5. In total, 27 NNs were fitted, and the best combination of hyper parameters was chosen using cross-validation. All models were trained with 20,000 epochs and a batch size of 256. An initial learning rate of 10^−3^ was used with a decay of 0*.*9 every 1000 epochs. In other words, the learning rate decreased by 10% every 1000 epochs.

For the classification task, the presence or absence of a parameter was predicted. The problem was reduced to the prediction of presence or absence (binary label simply named “model-label”) of nine model components: Mtt and Ktr, 1 order absorption, 0 order absorption, T_lag_, Fr, compartments 1–3, and linear and Michaelis–Menten elimination. Such reduction, shown in Fig. 3, resulted from combining parameters which existed only within the same model component (e.g., V2 and Q2 within the second compartment). A multi-label classification model was then trained on the training dataset derived according to this transformation. Two scenarios were studied. In scenario 1, the test set was created by randomly selecting 1600 observations from the whole dataset. The second scenario investigated the ability of the NN to predict a new model (i.e., a model that did not appear in the training set). To achieve this, all 400 observations of the two models were removed from the training set and used as the test set.

As for regression, different architectures of NN were investigated and cross-validated. The sigmoid function was chosen as the activation function. The loss was the cross-entropy loss. The number of epochs was set to 2000 with a batch size of 256. The same optimizer was used per the regression task. The learning rate was initially set to 10^−3^ with a decay of 10% applied every 200 iterations. For selecting the other hyper-parameters, a grid search was performed as summarized in Online Resource 6.

## Results

### PMX results

The results obtained with Monolix after estimation of the true models are summarized in Online Resource 7. PMX model selection followed the standard stepwise approach involving the selection of the structural model, assessment of the covariance matrix, and selection of the error model. Differently from ML methods, the model search did not include the exhaustive estimation of all models in the library for the five generated datasets, but it was based on decisions taken by the PMX modeler in classical stepwise fashion and according to common practice and criteria (mainly based on log likelihood, correlation and accuracy of parameter estimates, shrinkage). This aspect does not allow a direct comparison of the accuracy of the PMX model selection vs. ML methods. The two fitness functions for GA were based on the log-likelihood; therefore, a relative score was obtained.

### GA based model selection

GA assessment on the five true models was performed according to the two defined fitness functions, with and without the hybrid component and with two different generation sizes. Each model selection involved the evaluation of 400 models (20 generations with a population of size 20) without the hybrid component and approximately 280 models (11 generations with a population size of 20 and 15 models for each of the 4 local searches around the best models) with the hybrid component. In total, 20 model selections were performed. Table 4 presents the fitness of the best model from the last generation, along with its difference from the true model (Δ-fitness = fitness _GA selected model_ – fitness _true model,_ Online Resource 7), the GA runtime, and the selected model definition.

**Table 4:** Summary of GA-based model selection

Model #	True model	Selected model	Generation #	Shrinkage-related penalty	Hybrid	Runtime (h)	Fitness	Δ-fitness
1	1cmt, 1_abs, transit_cmt, lin_elim, prop_err	1cmt, 1_abs, lin_elim, prop_err	20	0	No	18.2	2370.6	– 65
		1cmt, 1_abs,, lin_elim, prop_err	11	0	Yes	11.1	2370.6	− 65
		1cmt, 1_abs,, lin_elim, prop_err	20	100	No	17.3	2376.5	− 150.7
		1cmt, 1_abs,, lin_elim, comb1_err	11	100	Yes	17.7	2376.7	− 150.5
2	1cmt, 1_abs, 0_abs, lin_elim, comb1_err	1cmt, 1_abs, lin_elim, comb2_err	20	0	No	22.8	2504.4	− 887.7
		1cmt, 1_abs, lin_elim, comb1_err	11	0	Yes	11.2	2505.7	− 886.4
		1cmt, 1_abs, lag, lin_elim, comb1_err	20	100	No	19.1	2522.2	− 856
		1cmt, 1_abs, lin_elim, comb2_err	11	100	Yes	10.7	2592.4	− 785.8
3	2_cmt, bolus, lin_elim, MM_elim, comb1_err	1_cmt, bolus, lin_elim, MM_elim, comb_err	20	0	No	16.2	1632.1	− 346.8
		1_cmt, bolus, lin_elim, MM_elim, comb1_err	11	0	Yes	14.2	1633.4	− 345.5
		1_cmt, bolus, lin_elim, MM_elim, comb2_err	20	100	No	17.3	1634.1	− 364.4
		1_cmt, bolus, lin_elim, MM_elim, comb2_err	11	100	Yes	15.6	1638.2	− 360.3
4	2_cmt, 1_abs, lag, lin_elim, add_err	1_cmt, bolus, lin_elim, add_err	20	0	No	20.5	4918.1	− 94.1
		1_cmt, bolus, lin_elim, MM_elim, prop_err	11	0	Yes	14.6	4928.4	− 83.8
		1_cmt, bolus, lin_elim, add_err	20	100	No	27.9	4921.5	− 201.4
		1_cmt, bolus, lin_elim, add_err	11	100	Yes	8.7	4921.5	− 201.4
5	3_cmt, bolus, lin_elim, comb1_err	1_cmt, bolus, lin_elim, comb1_err	20	0	No	14.2	2522.9	− 101.2
		1_cmt, bolus, lin_elim, comb1_err	11	0	Yes	12.2	2522.9	− 101.2
		1_cmt, bolus, lin_elim, comb1_err	20	100	No	18.1	2526.3	− 109.3
		1_cmt, bolus, lin_elim, comb1_err	11	100	Yes	9.5	2526.3	− 109.3

*Δ* delta, *GA* genetic algorithm, *h* hours, *1_cmt* one compartment, *2_cmt* two compartment, *3_cmt* three compartment, *1_abs* 1st order absorption, *0_abs* 0 order absorption, *lag* lag time, *transit_cmt* transit compartments, *lin_elim* linear elimination, *MM_elim* Michaelis–Menten elimination, *add_err* additive error model, *prop_err* proportional error model, *comb1_err* combined1 error model, *comb2_err* combined2 error model. GA selection was considered successful if the best model in the last generation (selected model) had a fitness value smaller than the true model (negative Δ-fitness)

As the selection criterion was not only based on the log-likelihood, but also included penalty terms (for parameters, shrinkage, and correlation), models without the highest likelihood or even simpler models could have been selected. Thus, GA selection was considered successful if the best model in the last generation had a fitness value smaller than the true model (negative Δ-fitness). The minimization of the fitness function is the main objective of the GA algorithms, which is why the definition of the fitness function is crucial, to ultimately obtain a satisfactory model. The results indicate that a negative Δ-fitness was achieved for most of the selected models.

Comparisons of results obtained with and without the hybrid component (Table 4) suggest differences only in computational cost. The use of different fitness functions led to differences in the statistical model in terms of correlations or distributions of random effects, but not to the structural and error models, which were the same.

For the first dataset, the best model across the four selections (with and without the hybrid component, for the two different fitness functions) was the 1 compartment model with 1 order absorption and linear elimination. The transitory compartment present in the true model was selected for some models of the last generation for the second fitness function. However, this was not the best model of the last generation, and thus it does not appear in Table 4. The selected error model was correctly predicted for three selections (fitness function 1 with and without hybrid component and fitness function 2 without hybrid component). For the second dataset, a 1 compartment model with 1 order absorption and linear elimination was selected. The mixed absorption (0 order and 1 order) present in the true model was not retained for the second dataset using GA. Depending on the number of generations (and the presence or absence of the hybrid component), two error models were selected: combined 1 (true) and combined 2. For the third dataset, the correct absorption routine and elimination routine were identified by all selections. The true error model (combined 1) was selected by three criteria. However, only 1 compartment instead of 2 was identified in all selections. For the fourth dataset, the selected model was far from the true one as the administration routine, lag time, and number of compartments, were not correctly identified by any of the four selections. Finally, with the second fitness function, the true fifth model was always correctly selected, except for the number of compartments (1 instead of 3).

### Neural network-based model selection

**Fig. 4:**
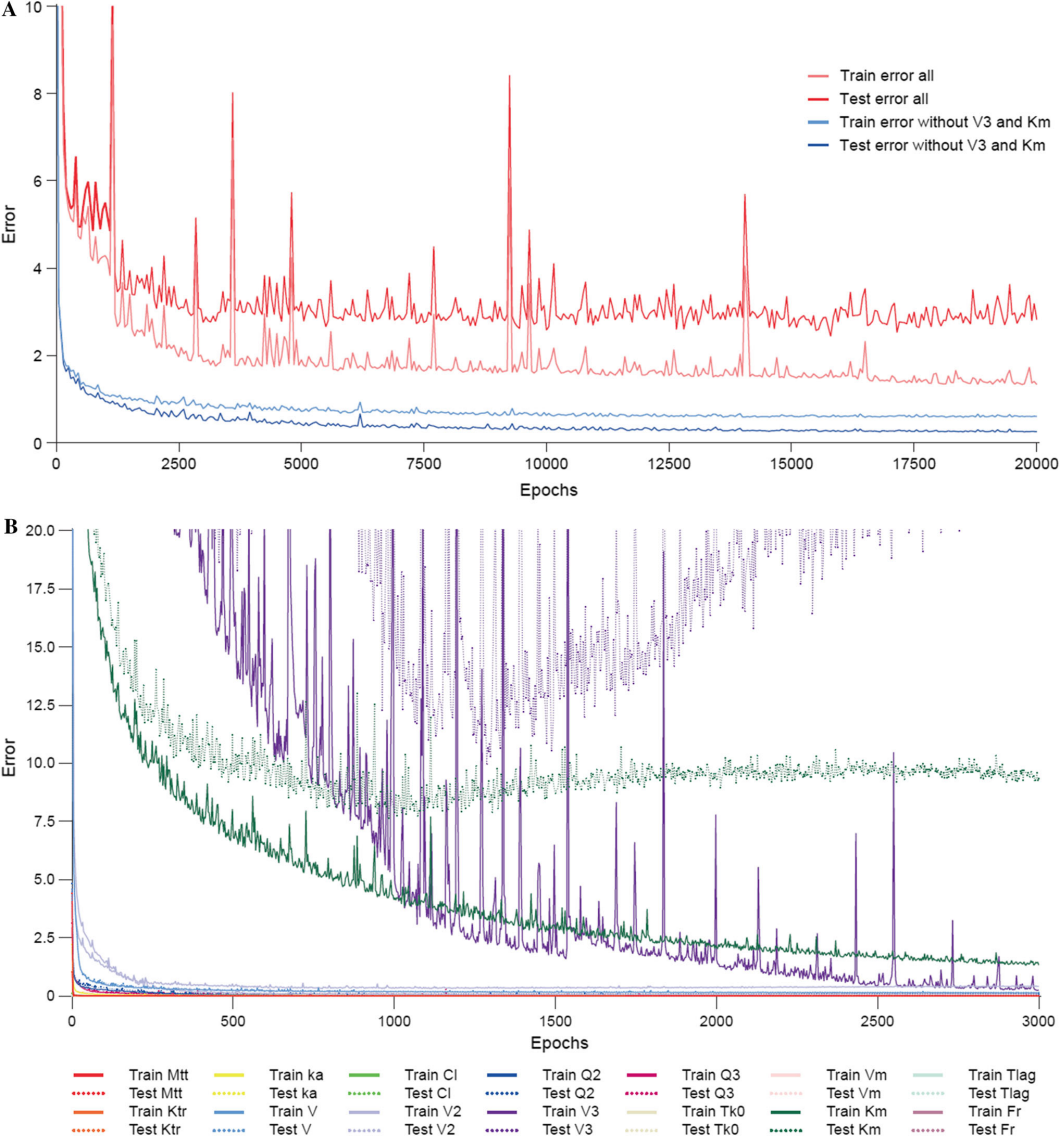
NN train and test MSE obtained for regression, **A** during the learning phase for the global NN, and **B** if 14 independent NN were trained for each of the parameters. On panel A, train and test MSE obtained during the learning phase for the global NN are shown in dashed and solid lines, respectively, for the full NN (red) and for the NN without prediction of Km and V3 (blue). On panel B, train and test MSE obtained during the learning phase are shown in dashed and solid lines, respectively, for 14 independent NN trained for each of the parameters. *CL* clearance, *Fr* bioavailability, *Ka* 1 order absorption, *Km* Michaelis–Menten elimination, *Ktr* transitory compartment, *Mtt* transitory compartment, *MSE* mean squared error, *NN* neural network, *Q2 and Q3* inter-compartmental clearance, *TK0* 0 order absorption, *T*_*lag*_ lag time, *V* volume for central compartment, *V2* volume for second compartment, *V3* volume for third compartment, *Vm* Michaelis–Menten elimination. *Note* Various NNs for regression were trained (Color figure online)

Some hyper parameters were defined before training the NN. The best architecture for regression used 10 hidden layers of size 50, with a weight decay of 10^−2^, and the grid-search reported in Online Resource 5. A summary of model parameter values in the generated data is reported in Online Resource 8. Frequencies of the different model’s components are also presented in Online Resource 9. The evolution of the test and train error (MSE) shown during the learning phase for regression is presented in Fig. 4a. Despite a decrease of the MSE during the training phase, a high final error equal to 1*.*7 for the training set and 2*.*8 for the test set (red curves) was obtained at the end of the training phase with the best networks. The large difference between these two errors suggests overfitting; therefore, additional investigations were performed.

Specifically, 14 independent and simple NNs (five hidden layers of size 25) were trained to assess the accuracy of predictions for each parameter. Figure 4b represents the evolution of the test and the training MSE for each network. The results indicate that two parameters (V3 and Km) were not correctly predicted. This failure might be explained by a lack of sufficient data generated from models including these parameters. For example, as displayed in Online Resource 9, out of the 20 models, only five (25%) had a third compartment (versus 65% including at least two compartments and 100% at least one compartment). Another reason could be that the architecture of the regression network was still too simple. To further assess the impact of the poor predictions for these two parameters on overall network performance, a global NN was trained to predict all parameters except V3 and Km. As shown by the blue curves in Fig. 4b, better accuracy in terms of train and test error was achieved, and the overfitting was no longer observed. This was further corroborated by train and test MSE values for each parameter (Online Resource 10).

**Fig. 5:**
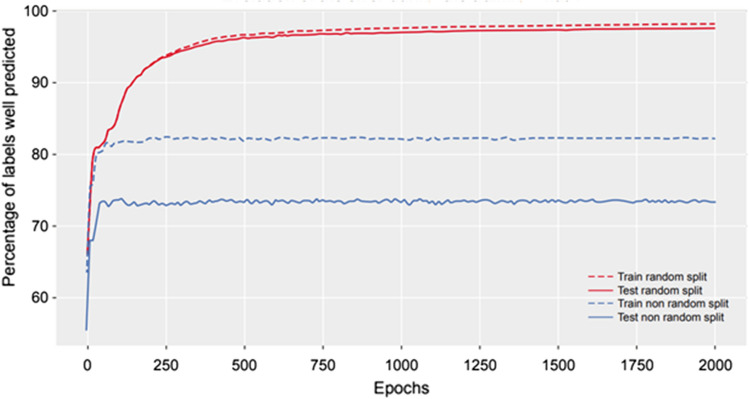
Evolution of the percentage of the label correctly predicted in the NN classification task. NN, neural network. Note: NN classification results are shown for scenario 1 (random split) where the test set was randomly selected (red curves), and for scenario 2 (non-random) where all observations of two models not included in the training set were selected to compose the test set (Color figure online)

Results from the classification task are presented in Fig. 5 for the best combination of hyper parameters: three hidden layers of size 30 with a weight decay of 10^−5^. Specifically, the evolution of the percentage of the label correctly predicted is shown. The curves show that the two NNs converge. For scenario 1, the NN achieved a training set accuracy of 97*.*9% and a final test set accuracy of 97*.*5%. The second scenario achieved a training set accuracy of 82.7% and a final test set accuracy of 73.8%. The differences between accuracies in the two scenarios and in particular, the lower number of model-labels correctly predicted in the second one, can be explained by one of the basic assumptions in ML, which is that the training and test sets should follow the same unknown distribution. In the second scenario, as all observations of two models were removed, this assumption does not hold.

## Discussion

ML models provide predictions on the outcomes of complex mechanisms by ploughing through databases of inputs and outputs for a given problem, but without necessarily capturing the nature of such processes [14]. Thus, adoption of these models should be fit-for-purpose and enabling the integration of interpretable output into more mechanism-based analytical methods [32]. The integration of ML into MID3 processes, especially PK/PD modeling, could confer benefits such as increased confidence in decision-making, more accurate predictions, and reduced time for model development, hence faster decision-making [2, 33, 34].

Classical model selection in PMX is often described as a linear process, starting with “structural” features, followed by random and covariate effects, each tested one at a time. In the optimization field, this is known as a “greedy” or local search algorithm. Like other greedy algorithms (e.g., the quasi-Newton used for parameter optimization), classical PMX approaches are at risk of arriving at local minima [33]. The ability of ML to leverage high-dimensional data and describe nonlinear relationships suggests ML may be better than classical PMX approaches [33].

The aim of this study was to investigate the ability of ML to perform population PK model selection. Four different approaches were used to recover the true models: PMX, GA, and NN with classification and regression. Model selection based on classical PMX approach was partly done manually while it was fully automated for ML (GA and NN) approaches.

GA is a global ML search method that may be a better alternative than classical PMX approaches [33]. GA creates a user-defined “search space” of candidate models representing all hypotheses to be tested, and uses this space to determine the optimal combination of “features” in a model. Initial experience suggests that GA consistently finds a better model than manual model selection [33]. In the current study we investigated GA models and classic PMX model approaches. Different settings of GA were tested, including a hybrid component and two fitness functions. GA selection was considered successful if the best model in the last generation had a fitness value smaller than the true model. The results indicate that a negative Δ-fitness was achieved for most of the selected models. The last generation of each selection showed that the best model appeared several times in the last generation, indicating GA convergence to a local or global minimum. However, even when the fitness of the selected model was less than the fitness criterion of the true model, GA tended to select models that were too simple, suggesting the penalties set for a new parameter may have been too high in this study or the simulated design not informative enough to enable selection of more complex models. Lower fitness values for GA selected models were mainly driven by the additional penalty (complexity and shrinkage) for datasets 1, 4 and 5 and by objective function differences for dataset 2 and 3 (Online Resource 11). As also noted in literature, it is commonly found that a model other than the "true" model may be "better" by any given criteria and then, a model selection algorithm cannot be judged based on whether it recovers the “true" model. Hence, model selection criteria need to be adjusted to be fit-for-purpose of each model (e.g., whether simpler model is preferred to a complex one, successful covariance step is required) and future extensions of this work should consider comparison of the predictive properties of the selected model versus the true simulations.

The NN ML approach may also confer benefits in PK modeling. Indeed, artificial NNs have been shown to confer advantages over traditional approaches (such as NONMEM), including increased accuracy and reduced variance [34]. Trained NNs were also able to correctly predict treatment effects across a range of doses whilst traditional regression provided biased predictions even when all confounders were included in the model [33]. In the current study, NNs with classification and regression were used to train the model using existing data. NN with classification demonstrated great ability to select models that appeared in the training set (i.e., models that were seen by the algorithm during the training phase). Although the training time was intensive, using a trained model on a new dataset to perform modelling did not require additional time. In contrast, the selection of hidden models (i.e., models that were not seen during the training phase) led to less accurate results. The NN for regression showed satisfying results; however, it is likely these could be improved by generating a larger training set. Indeed, the number of observations with non-0 values was too small for some parameters in the most complex models. These findings suggest the performance of the NN was strongly related to the size of the datasets in terms of number of (virtual) patients and the diversity of the models in the training dataset. The NN trained in this study was not able to accurately identify new models, likely due to the small size of the dataset. Despite the limitations of NN for regression in this study, this approach should be investigated in future with larger datasets. The library of PK models used in this work is a good representation of the true models describing the PK of the majority of compounds in literature. Further increasing the variety of models and the size of the training set will allow to increase the performances and the generalizability of NN. The model library may also be further extended to include additional complexities in terms of tested model structures and statistical models to include hierarchical variabilities. With the latter, the NN implementation would differ to account for inter-occasion variabilities of parameters as, for example, different instances from predicted parameter distributions. In summary, NN with classification can be used to select the structural model, followed by PMX software runs to fit the selected model. The trained NN methods can then be used to identify base models quickly for new compounds or drugs in development.

Computational costs were roughly equal for all models investigated but could not be directly compared. This is because PMX strategy cost depends on user validation, GA cost depends on user choice for the number of generations and the size of the generation, and because NNs do not require computation after the training (which in this study was within 1 day for regression and in less than 1 h for classification). However, computational costs required by ML methods suggested a significant benefit over traditional PMX procedures. A closer look at the GA runs showed that the first iterations required more time than the last ones. However, with average computational costs below 1 day, GA provided accurate model selections. Additionally, the use of the hybrid component in GA modeling reduced the computational cost by 34%. Of note, in our implementation, GA started with a random population of models with a first generation that can be “far” from the true model and then require increased computational costs, particularly if it seeks to estimate a complex model. A complete random generation is not mandatory in GA; optimization of initial parameters setting informed by existing knowledge could be explored in future work to constraint the generation around plausible models.

This work aimed at first establishing the proof of concept that ML can provide substantial benefit in terms of automation of the PK structural and random effect model selection. Further investigations would be needed to provide appropriate guidelines for its actual use. For instance, the impact of study design on models performance could be explored by considering real Phase I/II PK sampling schemes with investigation of multiple dosing and dose ranges. Representation of diverse data and PK models in the library would still be key to ensure generalizability. Future assessments should include the identification of suitable metrics for direct and automated methods comparison including the evaluation of typical PMX output (e.g., goodness of fit plots, relative standard errors) for GA as well as assessment of model predictive power.

Methodological workflows presented in the current work focused on the base model selection. While the optimization of covariate screening by using ML approaches can be addressed separately [21], both these model building steps could be combined by expanding GA and NN approaches to include the assessment of relationships between parameters and covariates. This could be done for GA by adding dimensions to the search space. For NN, a single prediction could be done to predict all features (structural, statistical, covariate) with a larger training set (in terms of individuals and in terms of features). Such network would be more complex than the one presented in this study and thus will require more data and computational power to be trained using the observed concentrations and any additional covariates.

## Conclusions

The use of ML in the pharmaceutical industry is in its infancy, with major advances anticipated in the coming years. In this new digital era, where increasing amounts of data are collected, integrating ML with PMX processes could confer great benefits within this discipline, including reduced computational costs and the ability to handle different data types without losing interpretability. The results of this study demonstrated that ML methods can greatly increase the efficiency of population model selection in case of large datasets or complex models requiring long run-times. Our results suggest that ML approaches can achieve a first fast selection which can be followed by more conventional PMX approaches. In addition, whilst we were unable to directly compare computational costs, our findings suggest costs are different between methods. NN requires a potentially time-consuming training step (although in this study this took less than 1 day); however, predictions can then be very fast. In this study, GA advised a model in less than 1 day. On the other hand, conventional PMX methods could take several days to weeks, depending on previous knowledge.

## Supplementary Information

Below is the link to the electronic supplementary material.

Supplementary file 1 (DOCX 371 kb)

## Data Availability

Any requests for data by qualified scientific and medical researchers for legitimate research purposes will be subject to the Merck KGaA, Darmstadt, Germany Data Sharing Policy. All requests should be submitted in writing to the Merck KGaA, Darmstadt, Germany data-sharing portal (https://www.merckgroup.com/en/research/our-approach-to-research-and-development/healthcare/clinical-trials/commitment-responsible-data-sharing.html). When Merck KGaA, Darmstadt, Germany has a co-research, co-development, or co-marketing or co-promotion agreement, or when the product has been out-licensed, the responsibility for disclosure might be dependent on the agreement between parties. Under these circumstances, Merck KGaA, Darmstadt, Germany, will endeavour to gain agreement to share data in response to requests.
