# Supplementary material for: Population pharmacokinetic model selection assisted by machine learning
Source: J Pharmacokinet Pharmacodyn. 2021 Oct 27;49(2):257–70. doi: 10.1007/s10928-021-09793-6 (PMC8940812; doi:10.1007/s10928-021-09793-6)
Supplement: Supplementary file 1 — (DOCX 371 kb) [file 10928_2021_9793_MOESM1_ESM.docx]

Journal of Pharmacokinetics and Pharmacodynamics

# Assessing the use of machine learning in pharmacokinetic model selection

Emeric Sibieude, Akash Khandelwal, Pascal Girard_,_ Jan S. Hesthaven, and Nadia Terranova

Correspondence: [nadia.terranova@merckgroup.com](mailto:nadia.terranova@merckgroup.com)

**Supplemental Material**

**Online Resource 1.** Design of simulated data.

PMX and GA were assessed on five generated datasets. For each true model, PK profiles were simulated for 200 virtual patients by using two time grids: [0, 2, 6, 20, 36] and [0, 4, 12, 24, 48] hours.

The full dataset used for NN comprised concentration profiles from 8000 virtual individuals generated according to the 20 different models reported in the table R1 below. A time grid of 48 time points taken every hour over two days was used for regression, while a reduced number of 24 time points (every 2 hours over two days) resembling a real scenario could be used for classification. From this, data from 1600 randomly selected individuals were removed from the learning phase and used for the test set.

Model parameters for all generated datasets were chosen by the scientist and checked for feasibility.

Table R1. Summary of simulated datasets investigated for creating the NN training and test sets

| Dataset | Input model | # Compartments | Output model | Error model |
| --- | --- | --- | --- | --- |
| Dataset 1 | Transit compartment + 1 order | 1 | Linear | Proportional |
| Dataset 2 | First order + 0 order | 1 | Linear | Combined 1 |
| Dataset 3 | Bolus | 2 | Michaelis-Menten + linear | Combined 1 |
| Dataset 4 | T_lag_ + 1^st^ order | 2 | Linear | Additive |
| Dataset 5 | Bolus | 3 | Linear | Combined 1 |
| Dataset 6 | 1^st^ order + 0 order | 1 | Linear | Proportional |
| Dataset 7 | T_lag_ + 1^st^ order | 1 | Linear | Additive |
| Dataset 8 | 1^st^ order | 2 | Linear | Proportional |
| Dataset 9 | T_lag_ + 0 order | 2 | Michaelis-Menten + linear | Proportional |
| Dataset 10 | Transit compartment + 1 order | 3 | Michaelis-Menten | Proportional |
| Dataset 11 | 0 order | 1 | Michaelis-Menten | Proportional |
| Dataset 12 | T_lag_ + 1^st^ order | 1 | Linear | Combined 1 |
| Dataset 13 | Transit compartment + 1 order | 2 | Linear | Combined 1 |
| Dataset 14 | 1^st^ order + 0 order | 2 | Michaelis-Menten + linear | Proportional |
| Dataset 15 | Bolus | 1 | Michaelis-Menten + linear | Combined 1 |
| Dataset 16 | T_lag_ + 1^st^ order | 2 | Michaelis-Menten + linear | Proportional |
| Dataset 17 | 0 order | 3 | Michaelis-Menten | Proportional |
| Dataset 18 | 1^st^ order | 3 | Michaelis-Menten + linear | Combined 1 |
| Dataset 19 | T_lag_ + 1^st^ order | 3 | Linear | Additive |
| Dataset 20 | 1^st^ order | 2 | Linear | Poportional |

**Online Resource 2.** GA example for a population of four chromosomes and translation of a model into a chromosome. **A** Population of four chromosomes (M1, M2 M3, M4) with the GA terminations. M5 and M6 are two possible children of M1 and M2. They are obtained by combining different genes of their parents. **B** Example of five-gene representation of one compartment model with one order absorption and linear elimination, with the error model represented by the last gene.


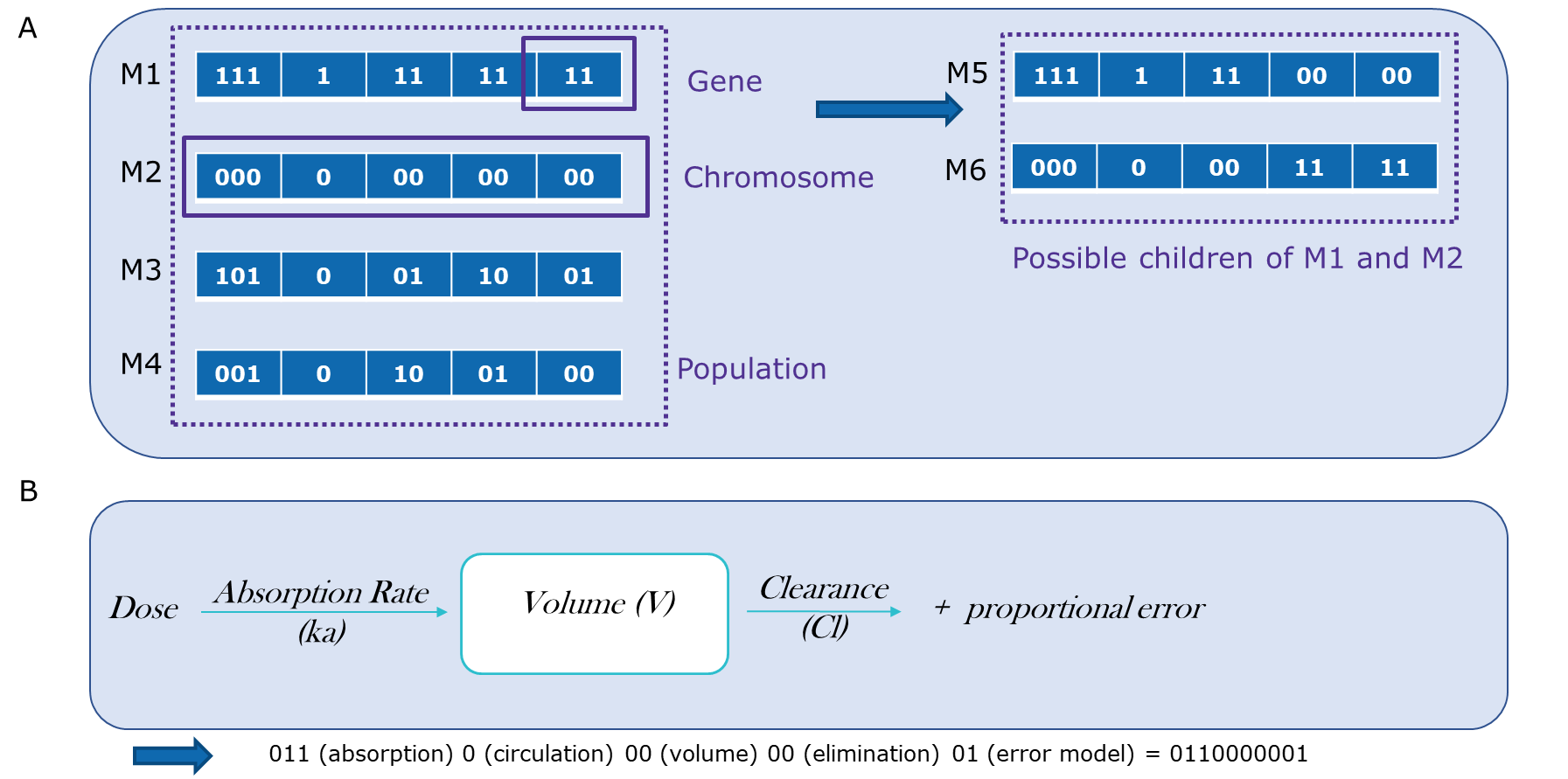


Cl, clearance; GA, genetic algorithm; ka, one order absorption; V, volume for central compartment.

**Online Resource 3.** Example of tournament selection for a population of four models in GA


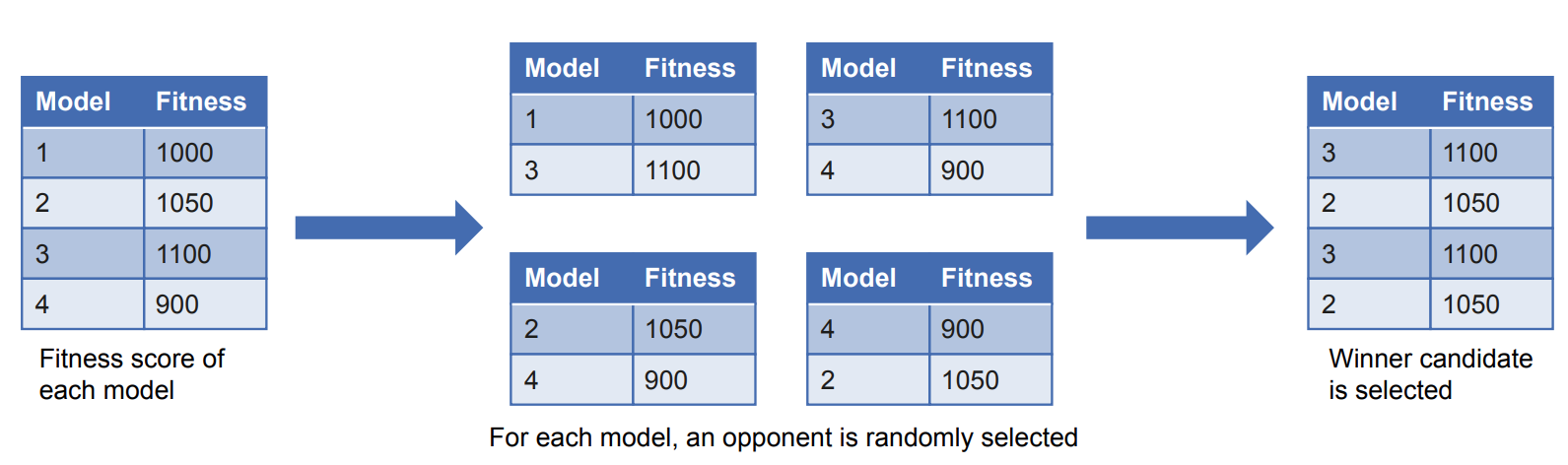


GA, genetic algorithm.

**Online Resource 4.** Example of crossover for one crossover location on the structural model in GA


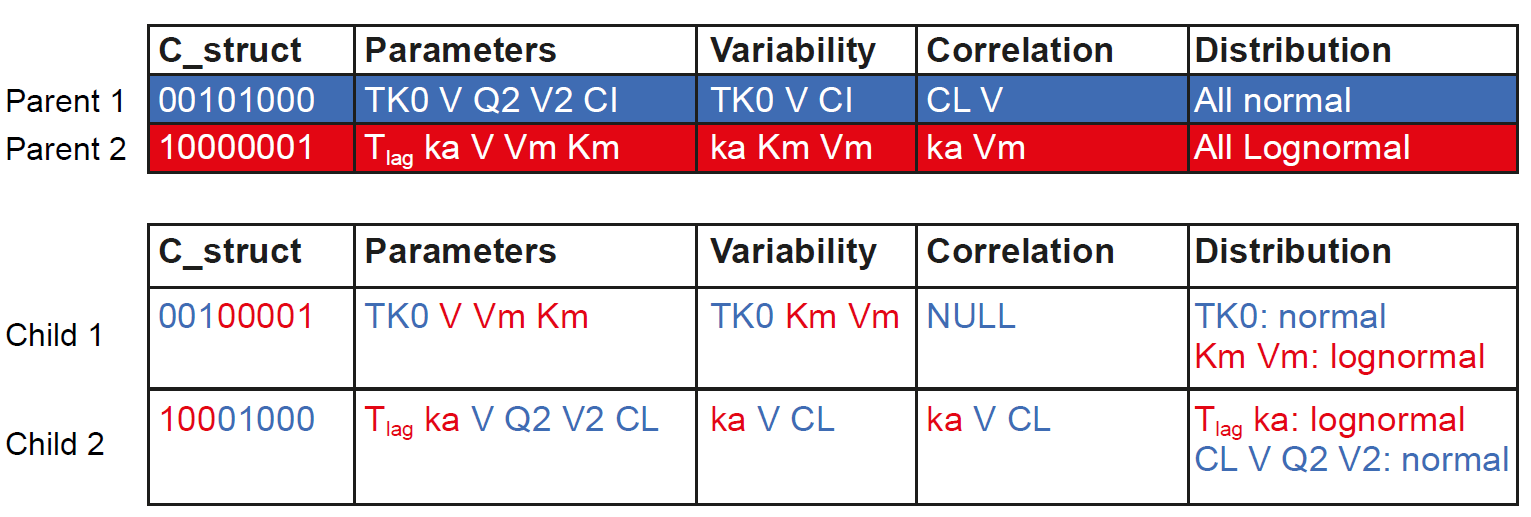


CL, clearance; GA, genetic algorithm; ka, one order absorption; Km and Vm, Michaelis-Menten elimination; Q2, inter‑compartmental clearance; TK0, 0 order absorption; T_lag_, lag time; V, volume for central compartment; V2, volume for second compartment.

Note: Heredities from parent 1 and parent 2 are shown in blue and red, respectively.

**Online Resource 5.** Summary of the grid search results for the NN regression task

| Weight decay | # Hidden layer | Hidden layer size | Validation error |
| --- | --- | --- | --- |
| 10−3 | 3 | 20 | 2*.*9 |
| 10−3 | 3 | 50 | 2*.*49 |
| 10−3 | 3 | 100 | 2*.*59 |
| 10−3 | 5 | 20 | 2*.*61 |
| 10−3 | 5 | 50 | 2*.*47 |
| 10−3 | 5 | 100 | 2*.*56 |
| 10−3 | 10 | 20 | 1*.*9 |
| 10−3 | 10 | 50 | 2*.*06 |
| 10−3 | 10 | 100 | 2*.*17 |
| 10−2 | 3 | 20 | 2*.*31 |
| 10−2 | 3 | 50 | 2*.*44 |
| 10−2 | 3 | 100 | 2*.*54 |
| 10−2 | 5 | 20 | 2*.*53 |
| 10−2 | 5 | 50 | 2*.*46 |
| 10−2 | 5 | 100 | 2*.*35 |
| 10−2 | 10 | 20 | 2*.*2 |
| 10−2 | 10 | 50 | 1*.*8 |
| 10−2 | 10 | 100 | 1*.*5 |
| 10−1 | 3 | 20 | 2*.*34 |
| 10−1 | 3 | 50 | 2*.*49 |
| 10−1 | 3 | 100 | 2*.*34 |
| 10−1 | 5 | 20 | 2*.*39 |
| 10−1 | 5 | 50 | 2*.*41 |
| 10−1 | 5 | 100 | 2*.*35 |
| 10−1 | 10 | 20 | 1*.*7 |
| 10−1 | 10 | 50 | 1*.*5 |
| 10−1 | 10 | 100 | 1*.*7 |

NN, neural network.

**Online Resource 6.** Summary of the grid search results for the NN classification task

| Weight decay | # Hidden layer | Hidden layer size | Validation error | Accuracy (%) |
| --- | --- | --- | --- | --- |
| 10−6 | 1 | 20 | 0*.*16 | 92*.*4 |
| 10−6 | 1 | 30 | 0*.*17 | 91*.*4 |
| 10−6 | 1 | 50 | 0*.*17 | 91*.*1 |
| 10−6 | 3 | 20 | 0*.*13 | 95*.*1 |
| 10−6 | 3 | 30 | 0*.*14 | 94*.*8 |
| 10−6 | 3 | 50 | 0*.*15 | 94*.*6 |
| 10−6 | 5 | 20 | 0*.*22 | 90*.*8 |
| 10−6 | 5 | 30 | 0*.*26 | 88*.*6 |
| 10−6 | 5 | 50 | 0*.*21 | 91*.*8 |
| 5 · 10^−6^ | 1 | 20 | 0*.*17 | 92*.*2 |
| 5 · 10^−6^ | 1 | 30 | 0*.*15 | 92*.*1 |
| 5 · 10^−6^ | 1 | 50 | 0*.*16 | 92*.*7 |
| 5 · 10^−6^ | 3 | 20 | 0*.*12 | 95*.*9 |
| 5 · 10^−6^ | 3 | 30 | 0*.*12 | 95*.*9 |
| 5 · 10^−6^ | 3 | 50 | 0*.*12 | 95*.*9 |
| 5 · 10^−6^ | 5 | 30 | 0*.*19 | 93*.*0 |
| 5 · 10^−6^ | 5 | 30 | 0*.*17 | 93*.*5 |
| 5 · 10^−6^ | 5 | 50 | 0*.*16 | 94*.*25 |
| 10−5 | 1 | 20 | 0*.*14 | 92*.*7 |
| 10−5 | 1 | 30 | 0*.*13 | 92*.*9 |
| 10−5 | 1 | 50 | 0*.*14 | 92*.*8 |
| 10−5 | 3 | 20 | 0*.*10 | 96*.*5 |
| 10−5 | 3 | 30 | 0*.*09 | 96*.*7 |
| 10−5 | 3 | 50 | 0*.*11 | 96*.*5 |
| 10−5 | 5 | 20 | 0*.*16 | 94*.*3 |
| 10−5 | 5 | 30 | 0*.*15 | 95*.*0 |
| 10−5 | 5 | 50 | 0*.*15 | 94*.*6 |

NN, neural network.

**Online Resource 7.** Summary of Monolix estimations of the true models using the PMX approach with different fitness functions

| Dataset | Objective function | Fitness function 1 | Fitness function 2 |
| --- | --- | --- | --- |
| Dataset 1 | 2325.6 | 2435.6 | 2527.2 |
| Dataset 2 | 2882.1 | 3392.1 | 3378.2 |
| Dataset 3 | 1838.9 | 1978.9 | 1998.5 |
| Dataset 4 | 4882.2 | 5012.2 | 5122.9 |
| Dataset 5 | 2484.1 | 2624.1 | 2635.6 |

PMX, pharmacometric.

**Online Resource 8.** Description of the generated data for the NN

| Parameters | Mtt | Ktr | ka | V | CL | V2 | Q2 | V3 | Q3 | TK0 | Vm | Km | T_lag_ | Fr |
| --- | --- | --- | --- | --- | --- | --- | --- | --- | --- | --- | --- | --- | --- | --- |
| Mean | 0.822 | 0.441 | 1.211 | 8.785 | 0.413 | 3.242 | 0.913 | 7.670 | 0.768 | 0.232 | 0.774 | 6.919 | 0.039 | 0.040 |
| SD | 2.421 | 1.241 | 1.486 | 4.916 | 0.389 | 6.270 | 2.203 | 23.990 | 1.780 | 0.407 | 1.564 | 11.582 | 0.112 | 0.104 |
| Min | 0.0 | 0.0 | 0.0 | 0.77635 | 0.0 | 0.0 | 0.0 | 0.0 | 0.0 | 0.0 | 0.0 | 0.0 | 0.0 | 0.0 |
| First quartile | 0.0 | 0.0 | 0.0 | 4.998 | 0.100 | 0.0 | 0.0 | 0.0 | 0.0 | 0.0 | 0.0 | 0.0 | 0.0 | 0.0 |
| Median | 0.0 | 0.0 | 0.971 | 7.192 | 0.267 | 0.984 | 0.113 | 0.0 | 0.0 | 0.0 | 0.0 | 0.0 | 0.0 | 0.0 |
| Third quartile | 0.0 | 0.0 | 1.709 | 13.686 | 0.793 | 2.009 | 0.732 | 0.144 | 0.012 | 0.177 | 0.356 | 11.380 | 0.009 | 0.0 |
| Max | 15.352 | 5.282 | 5.492 | 27.301 | 2.278 | 42.263 | 18.658 | 134.93 | 5.179 | 1.6467 | 8.656 | 68.333 | 0.658 | 0.4694 |

CL, clearance; Fr, bioavailability; ka, one order absorption; Km, Vm, Michaelis-Menten elimination; Ktr, transfer rate constant; Max, maximum; Min, minimum; Mtt, mean transit time; NN, neural network; Q2, inter‑compartmental clearance for 2^nd^ compartment; Q3, inter‑compartmental clearance for 3^rd^ compartment; SD, standard deviation; TK0, 0 order absorption ; T_lag_, lag time; V, volume for central compartment; V2, volume for second compartment; V3, volume for third compartment.

**Online Resource 9.** Frequencies of the different model’s components in the generated training and test data for NN


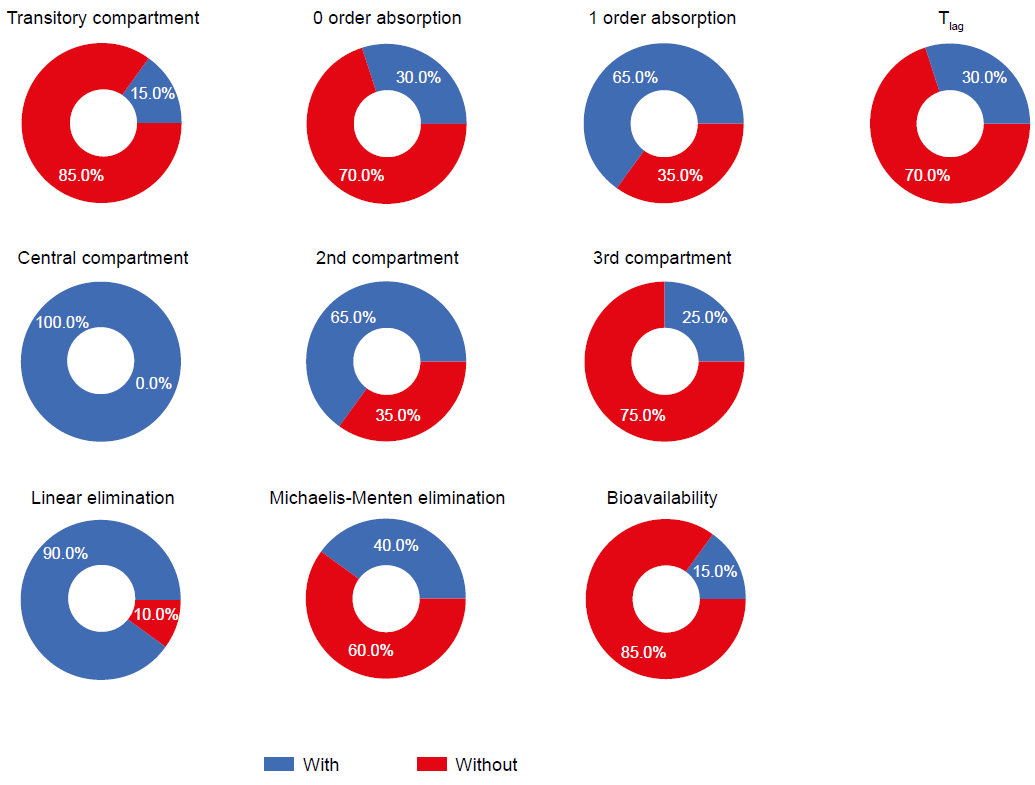


T_lag_, lag time.

**Online Resource 10.** Train and test MSE by parameter for regression.

|  | Mtt | Ktr | ka | V | Cl | V2 | Q2 | V3 | Q3 | Tk0 | Vm | Km | Tlag | Fr |
| --- | --- | --- | --- | --- | --- | --- | --- | --- | --- | --- | --- | --- | --- | --- |
| Train | 0,0128 | 0,0034 | 0,0180 | 0,1454 | 0,0083 | 0,0480 | 0,0149 | 0,2707 | 0,0023 | 0,0118 | 0,0950 | 1,3436 | 0,0011 | 0,0107 |
| Test | 0,0203 | 0,0034 | 0,0260 | 0,1693 | 0,0119 | 0,4263 | 0,0953 | 23,8809 | 0,0329 | 0,0171 | 0,1067 | 9,3628 | 0,0013 | 0,0112 |

**Online Resource 11.** Summary of differences in fitness terms between true models and GA selected models across considered fitness functions

| Model # | Objective function of GA-based model | Delta objective function | Delta parameter complexity | Delta shrinkage | Delta Non convergence |
| --- | --- | --- | --- | --- | --- |
| 1 | 2370.6 | -25  -25  -7.4  -18 | -40 | 0 | 0 |
|  | 2370.6 |  | -40 | 0 | 0 |
|  | 2376.5 |  | -43.3 | -100 | 0 |
|  | 2376.7 |  | -32.5 | -100 | 0 |
| 2 | 2504.4 | -457.7  -466.4  -464.3  -352.5 | -30 | 0 | -400 |
|  | 2505.7 |  | -20 | 0 | -400 |
|  | 2522.2 |  | -21.7 | 0 | -400 |
|  | 2592.4 |  | -32.5 | 0 | -400 |
| 3 | 1632.1 | -306.8  -305.5  -321.1  -321 | -40 | 0 | 0 |
|  | 1633.4 |  | -40 | 0 | 0 |
|  | 1634.1 |  | -43.3 | 0 | 0 |
|  | 1638.2 |  | -43.3 | 0 | 0 |
| 4 | 4918.1 | -24.1  -53.8  -25.9  -25.9 | -70 | 0 | 0 |
|  | 4928.4 |  | -30 | 0 | 0 |
|  | 4921.5 |  | -75.8 | -100 | 0 |
|  | 4921.5 |  | -75.8 | -100 | 0 |
| 5 | 2522.9 | -20.1  -20.1  -22.7  -22.7 | -80 | 0 | 0 |
|  | 2522.9 |  | -80 | 0 | 0 |
|  | 2526.3 |  | -86.6 | 0 | 0 |
|  | 2526.3 |  | -86.6 | 0 | 0 |

Delta of terms is calculated as difference between term of the true model and GA-based selected model.
